# App Technology to Support Physical Activity and Intake of Vitamins and Minerals After Bariatric Surgery (the PromMera Study): Protocol of a Randomized Controlled Clinical Trial

**DOI:** 10.2196/19624

**Published:** 2020-08-14

**Authors:** Stephanie Erika Bonn, Mari Hult, Kristina Spetz, Marie Löf, Ellen Andersson, Mikael Wiren, Ylva Trolle Lagerros

**Affiliations:** 1 Clinical Epidemiology Division Department of Medicine (Solna) Karolinska Institutet Stockholm Sweden; 2 Division of Upper Abdominal Surgery Karolinska University Hospital Stockholm Sweden; 3 Department of Surgery Linköping University Norrköping Sweden; 4 Department of Biomedical and Clinical Sciences Linköping University Norrköping Sweden; 5 Department of Health, Medicine and Caring Sciences Linköping University Linköping Sweden; 6 Department of Biosciences and Nutrition Karolinska Institutet Stockholm Sweden; 7 Department of Surgery Ersta Hospital Stockholm Sweden; 8 Center for Obesity Academic Specialist Center Stockholm Health Services Stockholm Sweden

**Keywords:** adults, body composition, exercise, metabolic health, obesity, randomized controlled trial, smartphones, vitamin intake, mobile phone

## Abstract

**Background:**

To optimize postoperative outcomes after bariatric surgery, lifestyle changes including increased physical activity are needed. Micronutrient deficiency after surgery is also common and daily supplementation is recommended.

**Objective:**

The aim of the PromMera study is to evaluate the effects of a 12-week smartphone app intervention on promotion of physical activity (primary outcome) and adherence to postsurgery vitamin and mineral supplementation, as well as on other lifestyle factors and overall health in patients undergoing bariatric surgery.

**Methods:**

The PromMera study is a two-arm, randomized controlled trial comprising patients undergoing bariatric surgery. Participants are randomized postsurgery 1:1 to either the intervention group (ie, use of the PromMera app for 12 weeks) or the control group receiving only standard care. Clinical and lifestyle variables are assessed pre- and postsurgery after 18 weeks (postintervention assessment), 6 months, 1 year, and 2 years. Assessments include body composition using Tanita or BOD POD analyzers, muscle function using handgrip, biomarkers in blood, and an extensive questionnaire on lifestyle factors. Physical activity is objectively measured using the ActiGraph wGT3X-BT triaxial accelerometer.

**Results:**

A total of 154 participants have been enrolled in the study. The last study participant was recruited in May 2019. Data collection will be complete in May 2021.

**Conclusions:**

Implementing lifestyle changes are crucial after bariatric surgery and new ways to reach patients and support such changes are needed. An app-based intervention is easily delivered at any time and can be a key factor in the adoption of healthier behavioral patterns in this rapidly growing group of patients.

**Trial Registration:**

ClinicalTrials.gov NCT03480464; https://clinicaltrials.gov/ct2/show/NCT03480464

**International Registered Report Identifier (IRRID):**

DERR1-10.2196/19624

## Introduction

### Background

The continuously increasing prevalence of obesity and its related comorbidities is a serious public health concern. Excess weight has been estimated to account for about 4 million deaths per year worldwide [1]. Bariatric surgery has been shown to result in marked sustainable weight loss and reduction of comorbidities in subjects with obesity [2,3]. Exercise interventions after bariatric surgery have been shown to provide additional weight loss and may be critical for long-term weight loss maintenance [4,5].

To optimize postoperative outcomes, lifestyle changes including increased physical activity are needed. Patients experiencing less weight loss, on average, 5 years after bariatric surgery have been shown to have lower levels of postoperative physical activity [6]. Micronutrient deficiencies are common in postoperative patients, despite the suggested use of routine vitamin and mineral supplements after surgery [7]. Low adherence to prescribed medication is a well-known cause of suboptimal effects in long-term treatment. Treatment with vitamin and mineral supplements after bariatric surgery has been associated with low levels of adherence [8]. With more than 5000 operations annually in Sweden alone, this is a growing group of patients in need of support to implement and maintain new habits [9].

Despite the known benefits of being physically active postsurgery, prolonged sedentary time and low levels of physical activity are common among patients having undergone bariatric surgery [10]. Objectively measured levels of physical activity postsurgery indicate that as few as 11% of patients reach the general recommendations of 150 minutes/week of moderate-to-vigorous-intensity physical activity 1 year after surgery [11]. Nevertheless, a systematic review and meta-analysis of 26 published studies suggests an increase in physical activity 1 year after bariatric surgery based on self-reported outcome measures [12]. Results from studies using objective tools to measure physical activity were, however, inconsistent. Patients are to a large extent inactive before surgery and do not seem to increase their physical activity much after [11,13]. New ways to promote and support postsurgery physical activity are therefore needed.

Woodlief et al [14] reported high compliance to, and increased levels of, physical activity and cardiorespiratory fitness after a 6-month-long exercise program among patients after bariatric surgery. However, such a program may not always be feasible in the clinic. Given the immense development of technology that has taken place during the past decades, mobile health (mHealth) (ie, medical or public health practice that is supported by mobile devices) [15] could be used to implement and aid in the adoption of healthier behavioral patterns in this continuously growing group of patients. Technology-based interventions for weight management have shown promising results and are easily disseminated to a large number of individuals [16].

With the rapid spread of smartphones during the past decade, apps are available for large-scale use. Previous studies using app-based strategies to target physical activity show improvements in behavior in different populations, including individuals with overweight and obesity [17]. The rate of smartphone possession in Sweden is over 90% in the adult population [18]. App-based interventions can be delivered anywhere, at any time, and provide opportunities for interactivity and individualized interventions.

### Aim

This paper describes the study design and methodology of the PromMera study, a randomized controlled trial using mHealth to promote physical activity and vitamin and mineral intake after bariatric surgery. The main aim of the trial is to evaluate the effect of a 12-week-long active intervention (ie, use of the PromMera smartphone app) on physical activity (primary outcome), as well as adherence to postsurgery vitamin and mineral intake and other lifestyle factors, including body weight, body composition, muscle function, eating behavior, health-related quality of life, sleep, and symptoms related to urinary incontinence or prolapse (secondary outcomes), compared to standard care.

### Hypothesis

We hypothesize that the intervention group using the PromMera smartphone app will have a higher level of physical activity, higher adherence to postsurgery vitamin and mineral intake, and improvement in other clinical and lifestyle factors after 12 weeks of active intervention (week 6-18 postsurgery), at long-term follow-up after 6 months, and 1 and 2 years postsurgery, compared to the control group.

## Methods

### Study Design and Recruitment

#### Overview

The PromMera study is a randomized, clinical, single-center trial with two parallel arms. Patients are recruited continuously at the surgical outpatient clinic at Vrinnevi Hospital in Region Östergötland, Sweden. The trial has been approved by the ethics committee of the Regional Ethical Review Board, Stockholm, Sweden.

Patients that are potentially eligible for bariatric surgery (ie, have a BMI ≥35 kg/m^2^) and who are referred to the surgical outpatient clinic are invited to attend a lecture where they receive information about bariatric surgery. After the lecture, patients who are still interested in having surgery are invited to go through a multidisciplinary evaluation for surgical eligibility in the outpatient clinic. During that visit, the patient receives both written and oral information about the PromMera study. Within a week, a nurse contacts all patients by phone to inform them if they are accepted for surgery or not. During this call, patients accepted for surgery are reminded about the PromMera study and offered participation; those who agree to participate give their oral informed consent.

A letter with further information about the study with a consent form to sign, a baseline questionnaire, an accelerometer to wear during 7 consecutive days, and a prepaid return envelope are sent to all patients that agree to participate. The signed consent form, the baseline questionnaire, and the accelerometer are returned to study personnel via mail. Participants are also contacted by study personnel and scheduled for presurgery assessment of body composition. Data on baseline measurements of height, weight, handgrip, body composition, and biomarkers measured in blood samples are collected at the visit in the outpatient clinic.

Postsurgery, all patients are prescribed lifelong supplementation of daily oral vitamin B12, calcium and vitamin D, and iron for menstruating women, in accordance with the Nordic Guidelines on Vitamin and Mineral Supplementation after Bariatric Surgery [19].

All patients are scheduled for a follow-up visit with a dietitian 6 weeks postsurgery. On this occasion, participants’ body composition and handgrip strength are measured. In the invitation letter to this visit, patients participating in the PromMera study are informed about their allocation to the intervention or control group. Participants in the intervention group are connected to the smartphone app during the meeting and can thereafter use the app during the following 12 weeks. All participants, both in the intervention group as well as in the control group, receive routine care postsurgery. The control group does not gain access to the PromMera app.

**Figure 1:**
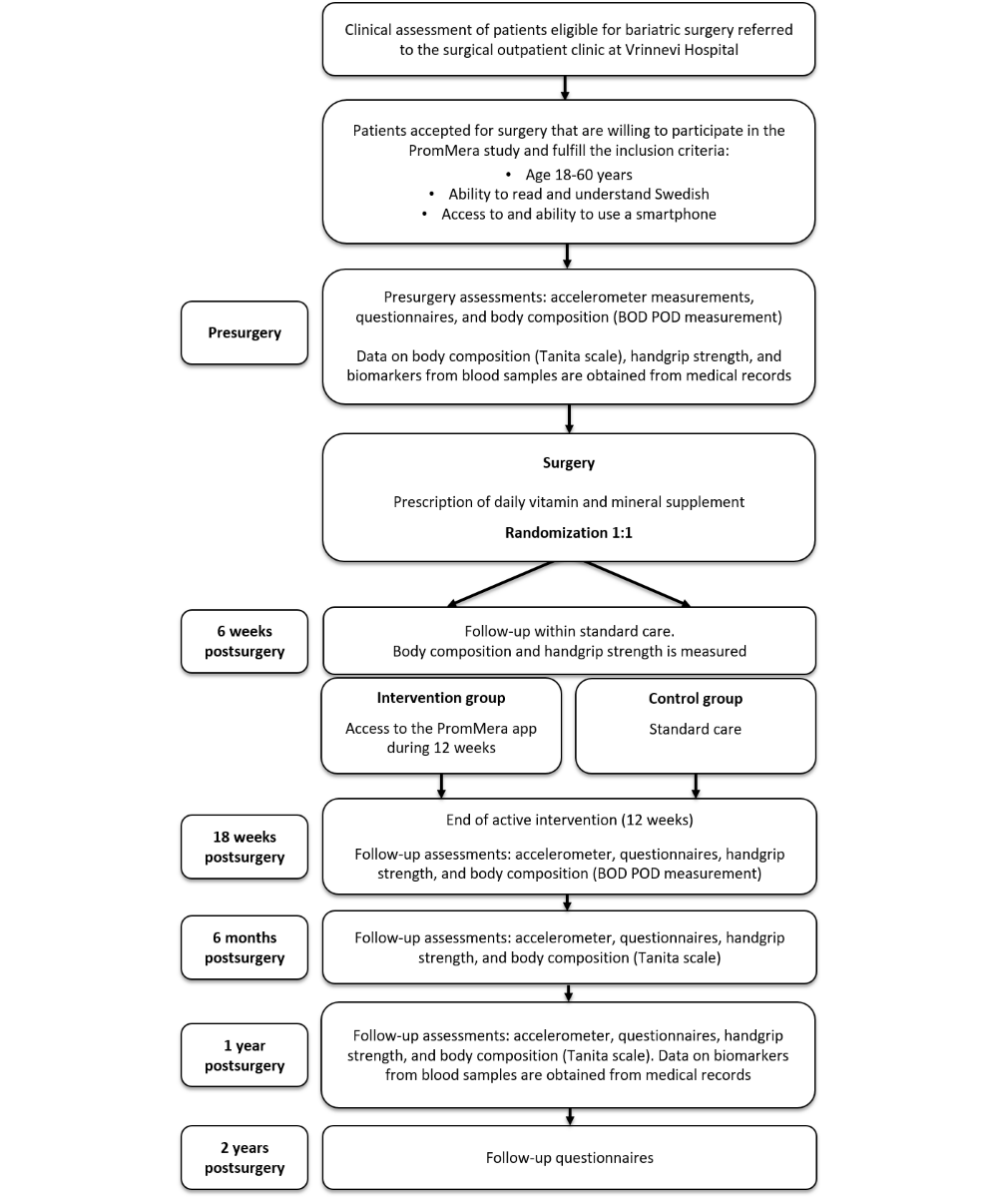
Flowchart of the PromMera study design.

Follow-up assessments are performed postsurgery at 18 weeks, 6 months, 1 year, and 2 years for all study participants. Assessments include accelerometer measurements, questionnaires, handgrip, and measurements of body composition. Body composition is measured using either the BOD POD (COSMED) or Tanita body composition analyzer. Participants are scheduled for additional follow-up assessments of body composition 18 weeks (BOD POD), 6 months (Tanita), and 1 year postsurgery (Tanita). Tanita measurements are performed within standard care while BOD POD measurements are an additional assessment performed by study personnel. Data on biomarkers from blood samples taken within routine care 12 months postsurgery are extracted from medical records. A flowchart of the study design is presented in Figure 1.

#### Inclusion and Exclusion Criteria

Patients that have been accepted for bariatric surgery and are between 18 and 60 years old are eligible for participation in the study. Participants must also be able to read and understand Swedish, have access to and the ability to handle a smartphone, and give their written informed consent prior to study start. Any disability preventing daily walking is an exclusion criterion.

#### Randomizing and Blinding

Study participants are randomized to either the intervention group (standard care and use of the PromMera smartphone app) or the control group (standard care). Randomization is done by study personnel after confirmation of the bariatric procedure at a 1:1 ratio by means of a random allocation sequence generated in an online program created for the study. Women and men are randomized separately in blocks of 4 and 2, respectively. The different block size is based on the uneven gender distribution in the patient cohort undergoing bariatric surgery, where approximately 75% are women [20]. Due to the nature of the intervention, it is not possible to blind the participants to their allocation.

### Intervention

#### Overall

The primary aim of the intervention (ie, use of the PromMera smartphone app) is to increase physical activity and adherence to postsurgery vitamin and mineral intake through use of the app during 12 weeks. The app is available both for iOS (compatible with version 8.0 and higher) and Android (version 4.1 and higher).

#### Download

In the invitation to the follow-up visit with a dietitian 6 weeks postsurgery, participants in the intervention group are instructed to download the PromMera smartphone app before attending the meeting. During the visit, participants receive a personal log-in from the dietitian and connect to the app. The active intervention starts the first Monday following the meeting and continues for 12 consecutive weeks.

#### The PromMera Smartphone App: General

In the PromMera smartphone app, the user sets a weekly goal for physical activity. The study participant is asked daily to register physical activity and intake of vitamin supplements. It is also possible, but not required, to register weight once a week. Within the app, information regarding physical activity, general information on medication use after surgery, vitamin and mineral supplementation, and diet recommendations is available in a library. Push messages encouraging the user to access and read the available information are sent according to a predetermined schedule, with more frequent messages during the first weeks of the intervention and less frequent messages toward the end. The app also includes contact information for clinical staff and study personnel.

#### The PromMera Smartphone App: Physical Activity

Each week, the participant sets a goal for the total number of minutes to be spent on an at least moderate level of physical activity during the coming week. Participants are able to choose a goal of 100, 150, 210, or 250 minutes. A notification to register the weekly goal is sent every Monday at noon.

Participants are asked to report all their activities of at least moderate intensity (minutes) in the app. When reporting activities of vigorous intensity, participants are instructed to double the minutes to account for the higher intensity. Minutes spent active can be registered continuously during the day (eg, a participant can report being active during 10 minutes on five different occasions during a day) or as a summarized total (eg, the same participant can choose to register 50 minutes at once at the end of the day). Activity minutes from previous days can be recorded afterward if needed. A daily reminder to register activity is sent every day at 8 PM.

The individual goal and total minutes reported per week are shown side by side in a graph. Participants can follow their progress each week and receive a feedback message each Sunday evening relating their reported minutes to their goal during the past week. If they have reached their personal goal and/or fulfilled the physical activity recommendation of at least 150 minutes of physical activity at a moderate-intensity level, they receive a message of encouragement to “keep up the good work” during the coming week. If they have not reached the goal, the message encourages them to try again next week.

#### The PromMera Smartphone App: Vitamin Intake

In the app, users are asked daily to respond to the question “Have you taken your vitamins today?” It is possible to report vitamin intake from previous days if needed. A reminder about registration of vitamins is sent together with the reminder of physical activity every evening. The participant can follow the personal recording of vitamins in the app where a bar chart displays green bars for days when the participant has reported taking the vitamins or red bars on days when no registration of vitamins has been done. A feedback message related to reported vitamin intake is sent out on Sundays at noon. A message saying “Well done, keep up the good work” is sent to all that have registered at least 6 days of vitamin intake. Those that have registered fewer days are encouraged to find new routines helping them to remember to take their vitamins the following week.

### Sample Size and Power Calculations

A total of 110 patients (55 in each group) provide 80% power at a 5% significance level to detect a 10 min/day difference in moderate physical activity (eg, 20 min/day versus 30 min/day), assuming an SD of ±18 min/day in both groups. To cover for a 20% dropout rate, we continued the recruitment process until more than 150 patients (75 in each group) with complete baseline data were included.

### Outcome Measures

#### Physical Activity

Physical activity, including sedentary behavior, is assessed using the ActiGraph wGT3X-BT triaxial accelerometer [21]. Accelerometer measurements are performed at baseline and postsurgery after 18 weeks, 6 months, 1 year, and 2 years. On each occasion, participants receive the accelerometer by mail together with a diary to record nonwear time. To increase feasibility and compliance with accelerometer use, participants are instructed to wear the accelerometer on the wrist during all hours for 7 consecutive days and to record all nonwear time (eg, when performing water activities or removing the accelerometer for other reasons) in the diary. A frequency of 80 Hz is used for data collection.

#### Body Composition

Information on weight, height, and body composition is measured at the hospital, both at the presurgery clinical examination (presurgery baseline assessment) and 6 months postsurgery for all patients. Height is measured to the nearest cm and weight is measured to the nearest 0.1 kg in light clothing. Body composition is measured using a Tanita digital body composition analyzer (Model DC-430MA), utilizing a 4-electrode bioelectrical impedance analysis with current going from the tips of the toes to the heels of both feet.

Body composition is further measured using the BOD POD (COSMED) body composition analyzer presurgery and at follow-up, 18 weeks postsurgery. Air-displacement plethysmography is used to measure body volume and, thereafter, body density is calculated using body weight and the measured body volume information.

#### Muscle Function

Muscle function is assessed by handgrip strength using a digital handgrip dynamometer (Takei 5401) [22]. Measurements are performed at the hospital at the presurgery clinical examination and after 18 weeks, 6 months, and 12 months postsurgery. During measurements, participants are standing upright and are instructed to relax while holding the dynamometer in their right hand alongside the body. Thereafter, they clasp the grip of the dynamometer with full force using their right hand first, then alternate left and right hands until two measurements have been done per side. Measurements are recorded to the nearest 0.1 kg and results from all four measurements are noted.

#### Biomarkers

Data on specified biomarkers are obtained from blood samples drawn and analyzed within routine care presurgery and 1 year postsurgery. Results from analysis of hemoglobin (g/L), s-ferritin (µg/L) or iron status (µmol/L), s-folate (nmol/L), p-calcium (mmol/L), s-albumin (g/L), s-25(OH)D (25‐hydroxyvitamin D) (nmol/L), p-parathyroid hormone (pmol/L), and s-cobalamin (pmol/L) are extracted from the medical records of study participants.

#### Registry Data on Supplement Use

The study participants are prescribed lifelong daily use of oral vitamin B12 as well as calcium and vitamin D, and menstruating women are prescribed additional iron. Patients are also recommended daily use of over-the-counter multivitamin tablets. Adherence rate to the prescribed supplements and persistence to treatment are measured from pharmacy refill data the first postoperative year. The pharmacy refill records are collected from the Swedish Prescribed Drug Register. The medication possession ratio is calculated and a patient is considered as nonpersistent if there is a gap in therapy longer than 180 days.

#### Questionnaire

Study participants respond to paper-based questionnaires at baseline (presurgery) and at follow-up after 18 weeks, 6 months, 1 year, and 2 years postsurgery. If not otherwise specified below, all questions are included in the baseline and four follow-up questionnaires. Each questionnaire takes approximately 30-45 minutes to complete.

*Background information* is assessed at baseline and 1 and 2 years postsurgery by questions on marital status, employment status, level of education, and tobacco use (ie, smoking and snuff use). Participants are also asked to report medical treatment, including the following: treatment for diabetes, hypertension, hyperlipidemia, depression or anxiety disorder, and joint pain or arthrosis, as well as CPAP (continuous positive airway pressure) treatment for sleep apnea. Missing information can be retrieved from medical records.

*Eating behavior* is assessed using the 21-item Three-Factor Eating Questionnaire (TFEQ-R21) comprising 21 questions [23]. Three categories of eating behavior are assessed: cognitive restraint (six questions), uncontrolled eating (nine questions), and emotional eating (six questions). A score is calculated for each behavior.

*Health-related quality of life *is assessed using the 36-item Short Form Health Survey (SF-36) comprising 36 questions (RAND Corporation). The questionnaire has been validated in Swedish populations [24,25] and is used in the large Swedish Obese Subjects (SOS) study [3]. The questionnaire measures health within eight different areas: physical functioning (10 questions), bodily pain (two questions), role limitations due to physical health problems (four questions), role limitations due to personal and emotional problems (three questions), emotional well-being (five questions), social functioning (two questions), vitality (four questions), and general health (five questions). An additional question on perceived change in health status is also included.

*Sleeping habits* are reported using six questions from the Karolinska Sleep Questionnaire [26,27]. Participants report the time of going to bed the previous evening, time of waking up in the morning thereafter, and time from going to bed to falling asleep. They are also asked to report how difficult it was to fall asleep (on a 5-step scale from not at all to very), the quality of sleep (on a 5-step scale from very good to very poor), and if they slept long enough (on a 5-step scale from yes, definitely enough to no, definitely too little).

*Neighborhood environment* is assessed using four questions adapted from the Neighborhood Environment Scale [28]. The questions ask about the neighborhood environment related to walking and include questions on accessibility, social environment, and safety.

*Symptoms related to urinary incontinence or prolapse* are assessed using six questions based on the International Consultation on Incontinence Questionnaire [29]. On a 4-step scale ranging from not at all to a lot, the participants respond to questions regarding frequency of urinating, leakage, and pain in the lower abdomen.

*Dietary intake* is assessed at baseline and 6 months, 1 year, and 2 years postsurgery using a semiquantitative food frequency questionnaire (FFQ) comprising 94 food items. The FFQ has been validated previously [30]. Participants report how often, on average, they consume included food items and beverages, including frequency and volume of alcohol consumption (ie, beer, wine, and liquor).

*Physical activity* is asked about using three general questions about time spent performing physical activities at a moderate-intensity level (at least) or higher and sitting time [31].

*Adherence rate* for vitamin and mineral supplementation is assessed at follow-up after 6 weeks, 18 weeks, and 1 year postsurgery using a Swedish translation of the 5-item Medication Adherence Report Scale (MARS-5) [32]. The 10-item Beliefs about Medicines Questionnaire (BMQ) Specific is used to study attitudes toward vitamin and mineral supplementation [33]

*Evaluation questions about the PromMera app* are included at the 18-week postsurgery follow-up for participants in the intervention group. Participants respond to 21 questions about the app. Use of the library feature in the app that includes information on physical activity and exercise, medication use, vitamin intake, and dietary intake; links to useful webpages; and contact information for study personnel are also assessed. Additionally, participants have the opportunity to leave a free-text comment about the app.

### Statistical Analysis

Collected data will be anonymized and continuously entered and stored at secure servers. Characteristics of the study participants will be described using descriptive statistics. Data will be checked for outliers and normality. The prespecified outcome measures assessed at follow-up at 18 weeks, 6 months, 1 year, and 2 years postsurgery will be compared between intervention and control groups. Differences between groups will be performed using *t* tests, analyses of variance (ANOVAs), and logistic regression. The intention-to-treat approach will be followed in the analysis. Long-term effects will be analyzed within and between intervention and control groups using data from the 1-year and 2-year follow-ups. Trends over time in outcomes will be analyzed using generalized estimation equations to assess the effect of both time and the intervention on outcomes. We will also test for statistical interaction effects between characteristics and the intervention. Analysis stratified by age, sex, and BMI will also be performed. Results from the evaluation of the app will be summarized using descriptive statistics and studied in association with baseline factors using linear and logistic regression.

### Ethics Approval, Consent to Participate, and Trial Registration

The trial was approved by the ethics committee of the Regional Ethical Review Board, Stockholm, Sweden (2016/1259-31/4; 2017/1406-32; 2017/2101-32). All study participants receive oral and written information about the study and give their written informed consent prior to study start. The trial was registered at ClinicalTrials.gov (NCT03480464).

## Results

The first study participants were recruited into the trial in November 2017. Data collection is ongoing. Enrollment of participants ended in May 2019. A total of 154 participants have been included. Data collection will be complete 2 years after the surgery of the last included study participant (ie, May 2021). The first findings from the study are expected to be published in 2021.

## Discussion

The PromMera study aims to evaluate the effect of a smartphone app intervention targeting postsurgery physical activity and vitamin intake in patients undergoing bariatric surgery. Modern technology has become a necessity in today’s society and could be a powerful tool in the clinical setting to support patients. However, new mHealth solutions must be scientifically evaluated before they can be implemented in health care.

Notable strengths of our study include the randomized controlled design, large sample size, and long-term follow-up. A priori calculations of statistical power and objective assessment of physical activity, anthropometric measures, as well as clinical variables are also strengths of this study.

A limitation to the study may be that all study participants were recruited from the same hospital in Sweden. However, patients undergoing surgery at this hospital were recruited from the entire region, including both rural and urban areas. They are similar to the general patient population undergoing bariatric surgery in Sweden regarding, for example, age and BMI [9]. Therefore, generalizability of results to the general population is reasonable. Nevertheless, the inclusion criteria of being able to read and understand Swedish may be a limitation, as patients with limited knowledge of Swedish are more prevalent in areas of lower socioeconomic status. The inclusion criteria of having a smartphone may also be a limitation. However, smartphone usage in Sweden is high (>90% of adults) and independent of socioeconomic status [18]. The PromMera app was developed for usage on both Android and iOS devices, which is a strength of the study, since most smartphone users can download and use it regardless of device. The fact that the control group receives standard care might impact compliance negatively if participants are disappointed with their group allocation. However, including a sham intervention in the physical activity setting, without encouraging the controls to become more physically active, would have been difficult and could potentially mask a true effect of the intervention.

A result of the marked weight loss following bariatric surgery is the reduction of comorbidities [2,3]. A recent meta-analysis of randomized controlled trials aiming to increase postoperative physical activity among patients undergoing bariatric surgery showed that patients receiving a postsurgery exercise intervention increased their levels of physical activity; they also experienced greater weight loss compared to those who did not receive the intervention [4]. Exercise interventions may also be critical for long-term weight loss maintenance [5]. Technology-based interventions for weight management [16], as well as app-based strategies to target physical activity [17], have been successful, but to our knowledge no app specifically targeting physical activity after bariatric surgery has been evaluated. An app-based intervention is easily delivered at any time and can be a key factor in the adoption of healthier behavioral patterns in this rapidly growing group of patients undergoing bariatric surgery.

## Abbreviations

ANOVA: analysis of variance
BMQ: Beliefs about Medicines Questionnaire
CPAP: continuous positive airway pressure
FFQ: food frequency questionnaire
MARS-5: 5-item Medication Adherence Report Scale
mHealth: mobile health
s-25(OH)D: 25-hydroxyvitamin D
SF-36: 36-item Short Form Health Survey
SOS: Swedish Obese Subjects
TFEQ-R21: 21-item Three-Factor Eating Questionnaire
